# Dysfunctional macrophages in Alzheimer Disease: another piece of the “macroph-aging” puzzle?

**DOI:** 10.18632/aging.101276

**Published:** 2017-08-05

**Authors:** Laura Costarelli, Marco Malavolta, Robertina Giacconi, Mauro Provinciali

**Affiliations:** Advanced Technology Center for Aging Research, Italian National Institute of Health and Science on Aging (INRCA), 60121 Ancona, Italy

**Keywords:** Alzheimer's disease, aging, monocytes/macrophages, phagocytosis, senescence

The concept of innate immune system as a “window” into the central nervous system (CNS) is increasingly accepted and accumulating evidence suggests an alteration of brain-immune system relationships as a potential pathological mechanism in Alzheimer's disease (AD). There are many unresolved issues in the different hypothesis of AD [1] and the hypothesis of a defective innate immune Aβ clearance by macrophages of AD patients [2] is a cornerstone to understanding AD pathogenesis. Surveillance by peripheral mononuclear phagocytes occurs both physiologically and during disease [3]. Circulating blood derived monocytes are able to infiltrate the brain and to remove cerebral Aβ deposits more effectively than resident microglia function, thus preserving tissue homeostasis [4]. The MCP-1/CCR2 axis seems to be crucial for monocyte chemotaxis and infiltration into the brain of APP/PS1 mice, as the depletion of CCR2 reduced the infiltration of these cells in the inflamed brain parenchyma [5]. In AD patients, Aβ phagocytosis and chemotaxis are impaired and monocytes showed poor differentiation into macrophages, reduced Aβ internalization into endosomes and lysosomes as well as abnormal expression of cyclooxygenase-2 and cytokines [2] and increased apoptosis. These observations raise at least three key questions: (1) Are the microglial cells senescent in AD? (2) Do the peripheral mononuclear phagocytes remove senescent dysfunctional microglia in physiological conditions? (3) Are peripheral mononuclear cells characterized by senescent dysfunctional changes in AD? Now, Hall and co-workers in an elegant study, have demonstrated a significant role of a new type of cells, p16(Ink4a)/β-galpH6-positive macrophages in aging, which previously was attributed solely to senescent cells (SCs). Human senescent fibroblasts implanted into the peritoneal cavity of SCID mice can attract innate immune cells that cause their rapid clearance. The use of alginate bead-embedded SCs prevents the immune attack and one of the major cell types attracted by secretory factors of SCs was a subpopulation of macrophages characterized by p16(Ink4a) gene expression and β-galactosidase activity (β-gal), thus mimicking the most typical properties of SCs.

Consistently, mice with p16(Ink4a) promoter-driven luciferase (p16^LUC^), developed bright luminescence of their peritoneal cavity within two weeks following implantation of SCs embedded in alginate beads. p16(Ink4a)/β-gal-expressing cells had surface bio-markers of macrophages F4/80 and were sensitive to liposomal clodronate used for the selective killing of cells capable of phagocytosis. The authors suggest that p16(Ink4a)/β-galpH6-positive macrophages subpopulation has the same right as SCs to be considered a possible contributor to aging. We have described a similar hypothesis underlying Alzheimer's Disease as the result of age-related dysfunctions in microglia and peripheral mononuclear phagocytes [6]. We focused on the possibility that senescent macrophages and microglia play an important role in AD onset or progression. Morphological alteration of the microglia described by Streit et al. [7] as “dystrophy” was for the first time identified as a senescent-associated change rather than as a marker of microglia activation. Dystrophic microglia appears to accumulate in humans with dementia, to colocalize with degenerating neuronal structures and to precede the spread of tau pathology in AD brains. Transcriptional profiling of Alzheimer blood mononuclear cells by microarray showed a dys-regulation in sporadic AD, of genes implicated in disruption of cytoskeletal integrity, endocytosis, lipid metabolism, DNA repair mechanisms and cellular defenses. The senescent expression profile in monocyte/macrophage cells in AD patients remains poorly understood. A better knowledge of this process could lead to novel approaches for the immunodiagnosis and therapy of AD. Some patients for example could benefit of immunomodulating treatments and aged peripheral macrophage functions may be considered as specific targets of senolytics in order to correct their dysfunctional features and, consequently, to reduce brain inflammation and Aβ clearance.
